# Liquid biopsy in hepatocellular carcinoma: circulating tumor cells and circulating tumor DNA

**DOI:** 10.1186/s12943-019-1043-x

**Published:** 2019-07-03

**Authors:** Qianwei Ye, Sunbin Ling, Shusen Zheng, Xiao Xu

**Affiliations:** 1 Division of Hepatobiliary and Pancreatic Surgery, Department of Surgery, the First Affiliated Hospital, School of Medicine, Zhejiang University, 79 Qingchun Road, Hangzhou, 310003 China; 2 NHC Key Laboratory of Combined Multi-organ Transplantation, Key Laboratory of the diagnosis and treatment of organ Transplantation, CAMS, Hangzhou, 310003 China

**Keywords:** Circulating tumor cells (CTCs), Circulating tumor DNA, Clinical application, Hepatocellular carcinoma, Liquid biopsy

## Abstract

Hepatocellular carcinoma (HCC) is one of the most common cancers and a leading cause of death worldwide. Due to latent liver disease, late diagnosis, and nonresponse to systemic treatments, surgical resection and/or biopsy specimens are still generally considered as the gold standard by clinicians for clinical decision-making until now. Since the conventional tissue biopsy is invasive and contains small tissue samples, it is unable to represent tumor heterogeneity or monitor dynamic tumor progression. Therefore, it is imperative to find a new less invasive or noninvasive diagnostic strategy to detect HCC at an early stage and to monitor HCC recurrence. Over the past years, a new diagnostic concept known as “liquid biopsy” has emerged with substantial attention. Liquid biopsy is noninvasive and allows repeated analyses to monitor tumor recurrence, metastasis or treatment responses in real time. With the advanced development of new molecular techniques, HCC circulating tumor cells (CTCs) and circulating tumor DNA (ctDNA) detection have achieved interesting and encouraging results. In this review, we focus on the clinical applications of CTCs and ctDNA as key components of liquid biopsy in HCC patients.

## Background

Hepatocellular carcinoma (HCC) is one of the most common cancers and a leading cause of death worldwide. There are more than 841,000 patients diagnosed with HCC globally. In China, HCC ranks third in cancer-related mortality [1] as the result of latent liver disease, late diagnosis, and limited treatments. Currently, surgical resection or liver transplantation remains the primary therapy for HCC patients. Unfortunately, most patients at the time of first HCC diagnosis, have already reached an advanced cancer stage, and only approximately 20–30% of the patients are eligible for surgical intervention. Moreover, although the 5-year survival rate of early HCC (BCLC stage A) is high (50–75%), the prognosis of HCC is still limited due to 50–70% recurrence rate after radical surgical resection or ablation [2]. Currently, early detection or monitoring HCC recurrence mainly relies on imageology, serum alpha-fetoprotein (AFP) levels and tissue biopsy [3]. Nevertheless, imaging and pathological examinations still have their limitations in diagnostic accuracy and sensitivity, while common serum markers display poor diagnostic performance [4]. Therefore, it is critical to find a robust method to detect early HCC and to monitor tumor recurrence.

A new diagnostic concept known as “liquid biopsy” has emerged with substantial attention over the past years [5, 6]. A liquid biopsy collects the sample of body nonsolid biological tissue, such as blood for different analyses. Several other body fluids could also be used for specific liquid biopsy applications, such as cerebrospinal fluid (CSF) for central nervous system tumors, saliva for head and neck tumors, pleural fluid for thoracic and metastatic cancers, ascites for abdominal and metastatic cancers, stool for gastrointestinal tract cancers and urine for urinary tract cancers [7]. Circulating tumor cells (CTCs) and circulating tumor DNA (ctDNA) are cornerstones of liquid biopsy. Besides, cell-free microRNA and extracellular RNA, such as exosomes or tumor-educated platelets (TEPS), are also present in a liquid biopsy specimen.

In HCC, the molecular pathogenesis is extremely complex and heterogeneous. Currently, the pathological profile of HCC is obtained from surgical or biopsy specimens. However, a conventional biopsy cannot always be performed routinely due to its invasive nature. The information acquired from a single biopsy provides a limited snapshot of a tumor and always fails to reflect its heterogeneity. However, a liquid biopsy can overcome this weakness, provide the genetic profile of all cancerous lesions (primary and metastatic tumors) as well as offer the opportunity to systematically and dynamically track genomic evolution [8]. Moreover, analysis of therapeutic targets and drug resistance-conferring gene mutations from CTCs and ctDNA released into the circulation contributes to a better understanding and clinical management of drug resistance in cancer patients. To date, excellent progress has been made using liquid biopsy as blood-based biomarkers in HCC. These novel biomarkers are believed to have great potential and could provide more detailed individualized decision-making during HCC management, including early detection, prediction of treatment and prognostic outcome. In this review, we focus on the clinical applications of CTCs and ctDNA as crucial components of the liquid biopsy in the HCC diagnosis, prognosis and therapy.

## Main text

### The biological basis of liquid biopsy

#### CTCs

**Fig. 1:**
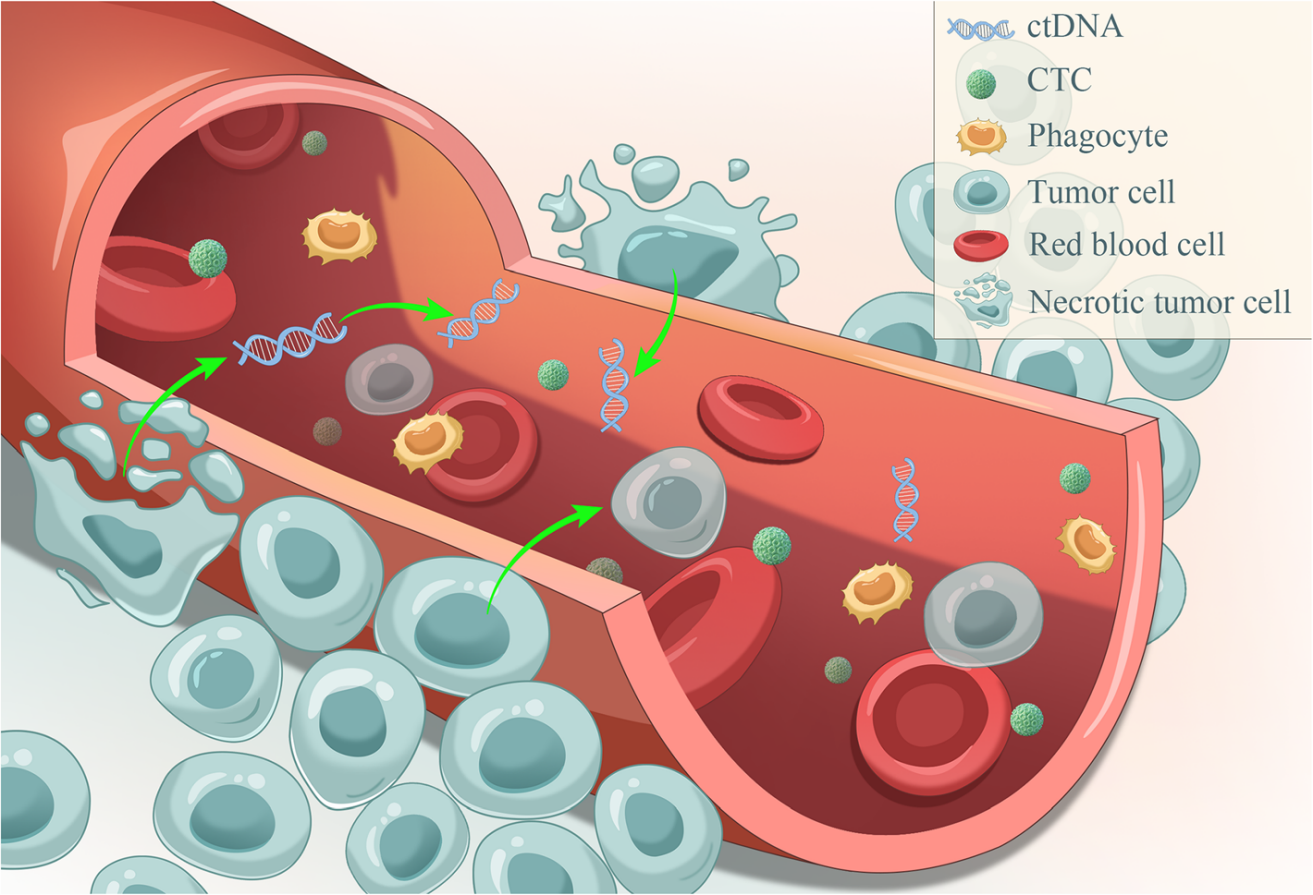
CTCs and ctDNA in the peripheral blood. CTCs and ctDNA are the essential elements and are widely believed to be the cornerstones of the liquid biopsy. CTCs are cancer cells that circulate in the bloodstream after being naturally shed from the original or metastatic tumors, they are “seeds” of tumors and can lead to a new fatal metastasis; ctDNA is derived from apoptotic and necrotic tumor cells that release their fragmented DNA into the circulation and contain genetic defects identical to the original tumor cells

In the 1860s, CTCs were discovered using a microscope to examine the peripheral blood [9]. In brief, CTCs are cancer cells that circulate in the bloodstream after being naturally shed from the original or metastatic tumors, which can lead to a new fatal metastasis and can be vividly described as “seeds” of the tumors (Fig. 1).

The number of CTCs in the blood are very low, which count up to a few hundred per milliliter depending on the available detection/isolation technology and the CTC definition used. To date, many “CTC” definitions exist; the CellSearch definition is considered as the current standard and states that a CTC is a circulating nucleated cell larger than 4 μm, expressing epithelial proteins EpCAM, and cytokeratins 8, 18, and/or 19, while being negative for the leukocyte-specific antigen CD45 [10].

The time of CTCs in the bloodstream is short (half-life: 1–2.4 h [11]), and the detection of CTCs in patient blood months or years after primary tumor resection indicates tumor recurrence or metastasis. Although the capture of CTCs from the whole blood is cumbersome, CTCs offer the opportunity to obtain information at the DNA, RNA and protein levels. However, the clinical application of CTCs remains challenging. The most important difficulty is that the earlier the cancer stage is, the fewer the CTCs. When CTCs enter the circulatory system, they will undergo apoptosis due to loss of adhesion to the extracellular matrix, hemodynamic shear forces, attacks of the body immune system [12] and target drugs. Only less than 0.01% of CTCs released into the circulation survive to produce metastasis [13]. These properties limit the application of CTCs detection in the early cancer diagnosis. Therefore, a detection technique of high sensitivity and specificity is urgently needed to expand the clinical applicability of CTCs.

#### ctDNA

Regarding ctDNA, cell-free DNA (cfDNA) must be mentioned. cfDNA is released by dying nonmalignant host cells and possibly also is actively secreted by lymphocytes [14]. Tumor DNA can be released from the primary or metastatic tumors, and CTCs into the blood of cancer patients [6]. It is widely accepted that the majority of such ctDNA is derived from apoptotic and necrotic tumor cells that release their fragmented DNA into the circulation [8, 14–16]; In principle, ctDNA contains genetic defects identical to the tumor cells they originated from (Fig. 1). However, ctDNA represents only a small fraction of the total cfDNA and is always diluted by larger quantities of DNA from noncancerous origins. Currently, there is no way to isolate ctDNA specifically from other circulating DNAs, and only the detection of tumor-specific mutations on circulating cfDNA indicates the presence of ctDNA [17].

cfDNA is double-stranded DNA existing in plasma or serum, and ctDNA molecules are usually shorter than nonmutant cfDNA molecules [18]. The modal size of ctDNA for many cancers is less than 167 bp, which is the length of DNA wrapping around the chromatosome. Additionally, selecting fragments between 90 and 150 bp improved the detection of ctDNA [19]. Interestingly, cancer patients have much higher levels of normal cfDNA than healthy individuals, which dilutes the ctDNA in particular when tissue-damaging therapies, such as surgery, chemotherapy, or radiotherapy, are administered [6]. Additionally, cfDNA has a short half-life between 16 min and 2.5 h [20, 21]. All these factors make ctDNA detection more difficult. Early studies showed that ctDNA possess many cancer-associated molecular characteristics, such as single-nucleotide mutations [22–26], methylation changes [27–30] and cancer-derived viral sequences [31–33], which may allow the discrimination of ctDNA from normal circulating cfDNA and guide the development of detection techniques.

### Methodology of liquid biopsy detection in HCC

In recent decades, CTCs and ctDNA have been intensively detected in HCC patients. Tables 1 and 2 summarize the previously demonstrated characteristics of CTCs and ctDNA.

**Table 1:** Variety of positive rate of CTCs in HCC

Region	HCC patients	Background liver status	Methodology	Positive rate	Ref.
China	139	HBV^a^: 85% LC^b^: 74%	CellSearch™ (Pre/post)	43.9 % 54.0%	Yu et al [34], 2018
China	112	HBV: 75%	CanPatrol	90.18%	Qi et al [35], 2018
Germany	57	CVH^c^:21% LC: 24% Alcohol: 7% NASH^d^: 10%	CellSearch™	15.8%	von Felden et al [36], 2017
United Kingdom	69	Alcohol: 32% NAFLD^e^:35% PBC^f^/AIH^g^:13% LC: 71%	ImageStream	65.2%	Ogle et al [37], 2016
China	36	Not applicable	CTC-Chip	100%	Zhang et al [38], 2016
China	42	HBV: 81%, HCV^h^: 2%, nonB, nonC: 17%	CTC-Chip	59.5%	Wang et al [39], 2016
United States	20	HBV: 25%, HCV: 45% HBV and HCV: 10% Alcohol: 5% NAFLD: 10%	CellSearch™	40.0%	Kelley et al [40], 2015
South Korea	81	HBV: 80%, HCV: 11%, Alcohol: 4% LC: 59%	RT-PCR^i^ (K19, CD44)	22.2%	Choi et al [41], 2015
Egypt	70	HCV: 100%	Flow Cytometry (CK19, CD133, CD90)	73% 69.5% 49.8%	Bahnassy et al [42], 2014
China	299	HBV: 90% LC: 90%	CellSearch™	42.6%	Guo et al [43], 2014
United Kingdom	52	Alcohol: 38%, HBV: 8% Diabetes: 12%	CellSearch™ ISET^j^	28% 100%	Morris et al [44], 2014
China	42	LC: 55%	CellSearch™	52.3%	Fang et al [45], 2014
China	27	Not applicable	CellSearch™	88.9%	Li et al [46], 2013
Germany	59	Alcohol: 38%, HBV: 17%, HCV: 13% LC: 89%	CellSearch™	30.5%	Schulze et al [47], 2013
China	123	HBV: 75% LC: 76%	CellSearch™	66.6%	Sun et al [48], 2013
China	60	HBV: 93% LC: 93%	Flow cytometry	50.0%	Liu et al [49], 2013
China	85	HBV: 84%, HCV: 7%, HBV and HCV: 5%, nonB, nonC: 4%	CellSearch™	81.0%	Xu et al [50], 2011
China	82	HBV: 80%	CellSearch™	68.3%	Fan et al [51], 2011
France	44	LC: 89%	ISET	52.2%	Vona et al [52], 2004
China	30	HBV:100%, LC: 100%	RT-PCR (MAGE1/3)	43.3%	Mou et al [53], 2002

^a^Hepatitis B Virus

^b^Liver cirrhosis

^c^Chronic viral hepatitis

^d^Non-alcoholic steatohepatitis

^e^Non-alcoholic fatty liver disease

^f^Primary biliary cirrhosis

^g^Autoimmune hepatitis

^h^Hepatitis C Virus

^i^Reverse transcriptase polymerase chain reaction

^j^Isolation by size of epithelial tumor cells

**Table 2:** Different targets of ctDNA in HCC

Region	HCC patients	Background liver status	ctDNA abnormalities methodology	Target	Ref.
United States	14	HBV^a^: 7.1%	Single nucleotide mutation	TP53, CTNNB1, PTEN, CDKN2A, ARID1A, MET	Ikeda et al [54], 2018
		HCV^b^: 50%			
		Alcohol: 14%			
			Amplification NGS^c^	CDK6, EGFR, MYC, BRAF, RAF1, FGFR1, CCNE1, PIK3CA	
Taiwan	237	HBV: 57.4% HCV: 29.5%	Methylation Pyrosequencing, Real-time PCR^d^	TBX2	Wu et al [55], 2017
Taiwan	180	HBV: 43% HCV: 15%	Methylation MS-PCR^e^	APC, COX2, RASSF1A	Lu et al [56], 2017
China	41	Alcohol: 34% LC^f^: 59% HBV: 92.7%	Single nucleotide mutation MiSeq™ system	TP53, CTNNB1, TERT	Liao et al [57], 2016
China	48	HBV: 81% LC: 83%	Single nucleotide mutation ddPCR^g^	TP53, CTNNB1, TERT	Huang et al [58], 2016
Taiwan	40	Not applicable	Methylation MS-PCR	HOXA9	Kuo et al [59], 2014
China	121	HBV: 83%	Methylation MS-PCR	MT1M	Ji et al [60], 2014
United States	66	HCV: 100% HCV and HBV: 6%	Methylation Pyrosequencing, MS-PCR	INK4A	Huang et al [61], 2014
China	160	HBV: 22%	Methylation MS-PCR	TRG5	Han et al [62], 2014
China	37	HBV: 100%	Methylation Bead Chip, Hot-start PCR, Pyrosequencing	DBX2, THY1	Zhang et al [63], 2013
China	43	HBV: 86%	Methylation MS-PCR	TFPI2	Sun et al [64], 2013
Italy	66	HCV: 51% Alcohol: 27%	Quantitative analysis Real-time PCR	hTERT	Piciocchi et al [65], 2013
Egypt	40	HCV: 100%	Methylation Real-time PCR	RASSF1A	Mohamed et al [66], 2012
Japan	220	HCV: 100%	Methylation MS-PCR	SPINT2, SRD5A2	Iizuka et al [67], 2011
China	72	HBV: 85%	Methylation MSRE-qPCR^h^	APC, GSTP1, RASSF1A, SFRP1	Huang et al [68], 2011
China	60	Not applicable	Quantitative analysis FQ-PCR^i^	hTERT	Yang et al [69], 2011
Egypt	28	HCV: 79% HBV: 18%	Methylation MS-PCR	APC, FHIT, P15, P16 E-cadherin	Iyer et al [70], 2010
China	130	Mostly HBV	Single nucleotide mutation RFLP^j^ and SOMA^k^	R249S (TP53 mutation)	Szymanska et al [71], 2009
China	19	HBV: 89%	Methylation MS-PCR	APC, GSTP1, RASSF1A, P16, E-cadherin	Chang et al [72], 2008
Hong Kong	85	HBV: 92%	Methylation RT-PCR^l^	RASSFIA	Chan et al [28], 2008
Taiwan	50	HBV: 22% HCV: 16%	Methylation MS-PCR	P15, P16	Zhang et al [73], 2007
Singapore	8	Not applicable	Methylation MS-PCR	RUNX3	Tan et al [74], 2007
China	79	HBV: 85% LC: 86%	Quantitative analysis Real-time PCR Allelic imbalance analysis	Not applicable D8S258 D8S264	Ren et al [75], 2006
Japan	52	HCV: 100%	Quantitative analysis Real-time PCR	GSTP1	Iizuka et al [76], 2006
Hong Kong	40	HBV: 83%	Methylation MS-PCR	RASSF1A	Yeo et al [77], 2005
Korea	46	HBV: 65% HCV: 22%	Methylation MS-PCR	P16INK4A	Chu et al [78], 2004
Hong Kong	49	Not applicable	Methylation MS-PCR	p16INK4a	Wong et al [79], 2003
Qidong, China	25	HBV: 84%	Single nucleotide mutation Direct sequencing	249^Ser^ p53 mutation	Huang et al [80], 2003
Hong Kong	25	HBV: 88% HCV: 2%	Methylation MS-PCR	P16	Wong et al [81], 2000

^a^Hepatitis B Virus

^b^Hepatitis C Virus

^c^Next-Generation Sequencing

^d^Polymerase Chain Reaction

^e^Methylation-specific PCR

^f^Liver cirrhosis

^g^Droplet Digital PCR

^h^Methylation-sensitive restriction enzymes-based quantitative PCR

^i^Real-time quantitative fluorescent PCR

^j^Restriction fragment length polymorphism

^k^Short oligonucleotide mass analysis

^l^Reverse transcription PCR

#### Technology platform for CTCs

In recent years, many different CTC isolation techniques have been developed, which can be generally divided into two groups: physical methods and biological methods. The former mainly depends on the physical properties of CTCs, such as size (filtration-based devices), density (Ficoll centrifugation), electric charge (dielectrophoresis), migratory capacity, and deformability [52]. The latter mainly relies on the antigen-antibody binding by using antibodies against tumor-specific biomarkers, including epithelial cell adhesion molecule (EpCAM), human epidermal growth factor receptor 2 (HER2), prostate-specific antigen (PSA) and so on [82]. Above all, EpCAM is the most commonly used antigen in CTC purification because its expression is virtually universal in cells of epithelial origin and is absent in blood cells [83]. Furthermore, the EpCAM-based CellSearch system is still the first and only clinically validated FDA-approved test for capturing and enumerating CTCs. Using anti-EpCAM-coated magnetic beads, CTCs are extracted from the blood, fixed, stained, and manually counted. However, this approach may miss highly metastatic potential tumor cells during the epithelial-mesenchymal transition (EMT) process, which is characterized by the decreased expression of epithelial markers and the acquisition of mesenchymal features [84].

In summary, multiple different approaches will likely be required to capture CTCs, possibly consisting of the combinations of less specific enrichment steps (physical methods), followed by more specific isolation techniques, including fluorescence in situ hybridization (FISH), microarray, immune-fluorescence, sequencing, flow cytometry, and reverse transcription polymerase chain reaction (RT-PCR) [85].

However, in liver cancers, only a small percentage (0–20%) of HCC patients were positive for EpCAM [86, 87]. As an alternative to macroscale systems, researchers have turned to emerging microfluidic technology to build promising microscale CTC isolation system since 2007, when a so-called CTC chip was developed to capture rare CTCs [88]. The CTC-chip has advantages, including enhanced interactions between the CTCs, functionalized surface and dynamic flows that prohibit nonspecific binding [89]. In summary, various types of techniques are available to collect and isolate CTCs, which will facilitate future cancer research. Of course, most techniques need to be validated as well.

#### Technology platform for ctDNA

ctDNA accounts for only a small percentage (sometimes < 0.01%) of the total cfDNA in the peripheral blood. The changes of ctDNA in plasma are quantitative and qualitative. The former refers to the total ctDNA concentrations, and the latter refers to DNA aberrations, such as single nucleotide mutations, copy number variations and methylation changes [85].

In recent years, many methods with high sensitivity and specificity have been developed, including digital PCR [90], BEAMing [21], Safe-SeqS [91], Capp-Seq [92] and TAm-Seq [93], to detect single-nucleotide mutations in ctDNA or whole-genome sequencing (WGS) to establish copy-number changes. In brief, techniques based on the analysis of ctDNA can be mainly divided as targeted or untargeted. The former aims to detect mutations in a set of predefined genes (e.g., KRAS), and the latter aims to screen the genome and discover new genomic aberrations (e.g., WGS), such as those confer resistance to a specific targeted therapy [94].

Currently, both digital PCR (dPCR) and next-generation sequencing (NGS) are the two advanced techniques for detecting DNA aberrations. The dPCR technique has been widely applied to detect targeted DNA aberrations. Although the PCR-based technique has very high sensitivity and, for example, can monitor tumor-associated genetic mutations at frequencies as low as 0.01% [22], this technique can only detect limited numbers of foci. To overcome this issue, the NGS technique is now used to obtain a more comprehensive view of the entire genomic landscape. Approaches involving deep sequencing include Safe-SeqS, Capp-Seq, TAm-Seq and AmpliSeq [95]. With these techniques, the NGS provides information to characterize personalized cancer gene maps and develop personalized medicine.

### Prognostic value of CTCs in HCC

CTCs are widely believed to be a significant determinant of metastasis and recurrence in cancers and are not recommended as an independent HCC diagnostic tool [96]. Therefore, CTCs may serve as a potential biomarker for prognosis.

Currently, EpCAM^+^CTCs have been intensively investigated in HCC, although the knowledge about their clinical relevance in HCC is lagging behind other major tumor types, such as breast cancer, prostate cancer and lung cancer [97]. The overall survival (OS) and disease-free survival (DFS) were significantly shorter in the CTC-positive cohort with HCC [34] and also associated with poor clinical characteristics [40]. However, the definition of CTC positivity is still conflicting. Some researchers defined CTC positivity as “≥1 CTC” or “≥2 CTC”, but others used “≥5 CTC” to analyze results. However, one thing for sure is that the more CTCs are detected, the poorer prognosis the patients will have.

In addition, EpCAM^+^CTCs may serve as a real-time parameter for monitoring HCC recurrence. A preoperative CTC (7.5 ml) of ≥2 is a novel predictor for tumor recurrence in HCC patients after surgery, especially in patient subgroups with AFP levels of ≤400 ng/mL or low tumor recurrence risk [48]. Nevertheless, only approximately 35% of HCC cases express EpCAM, limiting the clinical application of EpCAM^+^CTCs in predicting prognosis. Some other methods have tried to overcome this dilemma. For instance, detection of EpCAM+CTCs with co-existing CD4 + CD25 + Foxp3+ Treg cells indicated HCC recurrence. Zhou et al. [98] discovered that patients with high CTC/Treg levels showed a significantly higher risk of developing postoperative HCC recurrence than those with low CTC/Treg levels (66.7% vs. 10.3%, *P* < 0.001). Apart from this attempt, other subtypes of CTCs for early prediction of HCC recurrence were also explored. Zhong et al. [99] found that of the 62 HCC patients, mesenchymal CTCs and mixed CTCs in the recurrence group were significantly higher than in the nonrecurrence group, and mesenchymal CTC positivity (HR = 3.453, *P* = 0.007) was an independent risk factor for early recurrence. A similar study [35] also found that CTC count ≥16 and mesenchymal-CTC percentage ≥ 2% before resection were significantly associated with early recurrence, multi-intrahepatic recurrence, and lung metastasis. Altogether, different markers and combinations in CTCs can serve as a tool for predicting HCC recurrence and prognosis, but those markers may not be specific to HCC and limit the use of CTCs in early diagnosis in some way.

### Diagnostic value of ctDNA in HCC

In general, ctDNA mainly shows great diagnostic value in HCC. ctDNA is superior to that of previously described plasma biomarkers in terms of higher sensitivity and better clinical correlation.

In recent years, the “methylation pattern” of ctDNA has been the most intensively investigated hotspot [13]. Since methylation changes in ctDNA occur early in tumorigenesis and are potentially reversible, changes in methylation may offer the best hope for early cancer detection. Furthermore, methylation patterns are unique to each cell type and are highly stable under physiologic or pathologic conditions [100]. Therefore, the recognition of different methylation patterns may have the potential to serve as discriminatory tools for the detection and diagnosis of HCC. Wong and colleagues [81] detected for the first time that concurrent p15 and p16 methylation was positive in the plasma/serum of 92% (11 of 12) of HCC patients. Following this study, many researchers investigated the cfDNA methylation profile in HCC patients. For instance, Ras association domain family 1A (RASSF1A) promoter hypermethylation [77] was detected in 90% of the HCC group and could differentiate HCC patients from healthy controls and chronic HCV infection alone with an overall predictive accuracy of 77.5 and 72.5%, respectively. Therefore, aberrant promoter methylation in ctDNA could be evaluated as a screening tool for HCC patients, especially for small HCC among high-risk populations at an early stage.

Moreover, several hot methylated genes were combined to diagnose HCC. For example, p16, p15 and RASSF1A were explored in the serum of 50 HCC patients and provided an overall predictive accuracy of 89% with sensitivity and specificity of 84 and 94%, respectively [73]. A panel of four genes (APC, GSTP1, RASSF1A, and SFRP1, [68]) could discriminate HCC from normal controls with 92.7% sensitivity and 81.9% specificity. Additionally, three abnormally methylated genes (APC, COX2, RASSF1A) and one miRNA (miR-203) were combined to establish a predictive model by which nearly 75% of patients, who could not be diagnosed with AFP at 20 ng/mL, were detected [56]. Recently, Xu et al. [101] identified an HCC-specific methylation marker panel including ten markers and constructed a diagnostic prediction model that showed high diagnostic specificity (90.5%) and sensitivity (83.3%) superior to AFP (AUC 0.696 vs. 0.816), differentiating HCC patients from those with liver diseases or healthy controls. Interestingly, normal controls with positive detection likely to had etiological risk factors of HCC such as HBV infection and/or alcohol drinking history. In conclusion, hypermethylation in promoter regions is now recognized as an important early event in carcinogenesis. The combinations of different methylated tumor suppressor genes were absent or very low in normal tissues DNA. Therefore, detection of those DNA is specific to HCC and can diagnose HCC with high specificity and sensitivity.

Currently, the most commonly used serum marker AFP has limited diagnostic value because of low sensitivity of only 50% in HCC. cfDNA levels could discriminate HCC and HCV carriers at the optimal cutoff value of 73.0 ng/ml with a sensitivity of 69.2% and a specificity of 93.3% [76]. In addition, the combined AFP and cfDNA detection can improve HCC diagnosis. A study [102] showed an elevated diagnostic value with 95.1% sensitivity and 94.4% specificity in discriminating HCC from normal controls when using the combined detection of cfDNA and AFP. A similar study [103] found that the sensitivity of the combined detection of cfDNA with one marker (AFP or AFU) and cfDNA with two markers (AFP and AFU) was 71.8, 87.2 and 89.7% vs. 56.4, 53.8 and 66.7% for cfDNA, AFP and AFU when used alone, respectively. In AFP-negative HCC, the levels of plasma hTERT DNA in HCC patients with AFP ≤ 20 ng/ml were significantly higher than in HBV patients [69], indicating that hTERT DNA may serve as a novel complementary tool for AFP in the screening and detection of HCC. In a word, quantitative analysis of cfDNA is sensitive and feasible, and the diagnostic value of cfDNA is superior to AFP or AFU. Combined detection of cfDNA with AFP or AFU or both could improve diagnostic sensitivity of HCC. Furthermore, it is intriguing to imagine quantitation of cfDNA together with somatic mutation analysis to screen malignancy in the further. However, the definition of elevated cfDNA level is hard to decide and the optimal cut-off point is controversial in different researches because of various detection methods used.

Apart from its association with the malignancy, cfDNA could also be used as a potential biomarker to stratify liver fibrosis in patients with non-alcoholic fatty liver disease (NAFLD). Hardy, T. et al. [104] found that plasma DNA methylation of the peroxisome proliferator-activated receptor gamma (PPARγ) gene promoter increased with fibrosis severity and could be used as an independent predictor of fibrosis severity in NAFLD.

### Prognostic value of ctDNA in HCC

Except for early diagnosis, ctDNA also plays a vital role in HCC prognosis. For untargeted ctDNA, the levels of cfDNA are always negatively associated with DFS and OS [105, 106] and may serve as an independent prognostic factor for HCC recurrence and extrahepatic metastasis [107].

Targeted ctDNA detection can also reflect intratumoral heterogeneity and predict poor prognosis. Hot spot mutants, such as TP53, CTNNB1, and TERT, were the most preferred targets for the detection of ctDNA aberrations. Using droplet digital PCR, 27 of 48 HCC patients (56.3%) were found to have at least one kind of circulating mutant, with the mutant allele frequency ranging from 0.33 to 23.7% [58], indicating intra-tumoral heterogeneity in HCC. Another study used the MiSeq™ system to detect the same mutations, finding that ctDNA with mutations could be detected more easily in patients who suffered vascular invasion (*P* = 0.041) and predicted a shorter DFS time (*P* < 0.001, [57]). In addition, allelic imbalance (AI) on chromosome 8p at D8S264 in circulating plasma DNA was closely associated with the 3-year DFS (*P* = 0.014), and combined detection with cfDNA levels was independently associated with DFS (*P* = 0.018) and OS (*P* = 0.002) of patients with HCC [75]. In general, continuous detection of tumor-associated mutations in ctDNA can overcome the limitations of tumor heterogeneity and predict prognosis of HCC. However, there is still a problem because the circulating mutants are not all derived from tumor cells since germline mutation might also be detected in plasma. Therefore, it is necessary to screen corresponding tumor samples to identify their origins.

### Tumor monitoring and therapeutic evaluation

Surgical resection remains the primary treatment for HCC. ctDNA and CTCs are also used to assess the effect of surgical resection and monitor tumor burden. For instance, Wong et al. [79] found that the median p16INK4a methylation induced in plasma and buffy coat concordantly decreased 12- and 15-fold after surgical resection, respectively; Fan and his colleagues [108] used an orthotopic model by in vivo flow cytometry to detect CTCs in HCC, finding that the number of CTCs and early metastases decreased significantly after the resection, concluding that the resection prominently restricted hematogenous disseminating and distant metastases.

Subtypes of CTCs may associate with therapeutic response and could serve as a supplement for molecular subtype in HCC. Nel et al [109] investigated 11 patients with different therapies (watch and wait: 3; selective internal radiation therapy: 5; transarterial chemoembolization (TACE): 1; resection: 1; Nexavar: 1), detected a remarkable variation of CTCs with epithelial, mesenchymal, liver-specific, and mixed characteristics and found that different subgroups varied significantly among patients. Importantly, these CTC subgroups were associated with the therapeutic outcomes, and an increase in epithelial cells was associated with a worse treatment outcome in HCC patients. In addition, pERK+/pAkt- CTCs were more sensitive to sorafenib and remained an independent factor associated with a good prognosis (hazard ratio = 9.389, *P* < 0.01) in HCC patients [110]. Moreover, in vitro released CTCs were also evaluated for their sensitivity to chemotherapeutic drugs. Zhang *at el* [38] used a microfluidic chip to isolate and release viable CTCs, and the CTCs-treated sorafenib (5 samples) or oxaliplatin (5 samples) formed a significantly less number of spheroids compared to the control. To conclude, individual profiling of CTCs may have distinct clinical implications, which might help to predict outcome and potentially to select the appropriate treatment.

Importantly, somatic mutation frequency of ctDNA reflected clinical dynamics corresponding to sequential therapy and might provide a possible solution for monitoring tumor burden and prognosis. Cai et al. [111] monitored an HCC patient during the course of comprehensive therapy and observed an increased circulating level of 8 somatic mutations even before imaging diagnosis and the increase of AFP levels after the first TACE treatment. In this process, 1 nonsynonymous somatic mutation (HCKp. V174 M) was identified after the first TACE treatment and then became undetectable after the second surgery, while it sharply increased during the second recurrence. Therefore, ctDNA could track the change of different mutants and therapeutic responses in real-time longitudinal monitoring.

**Fig. 2:**
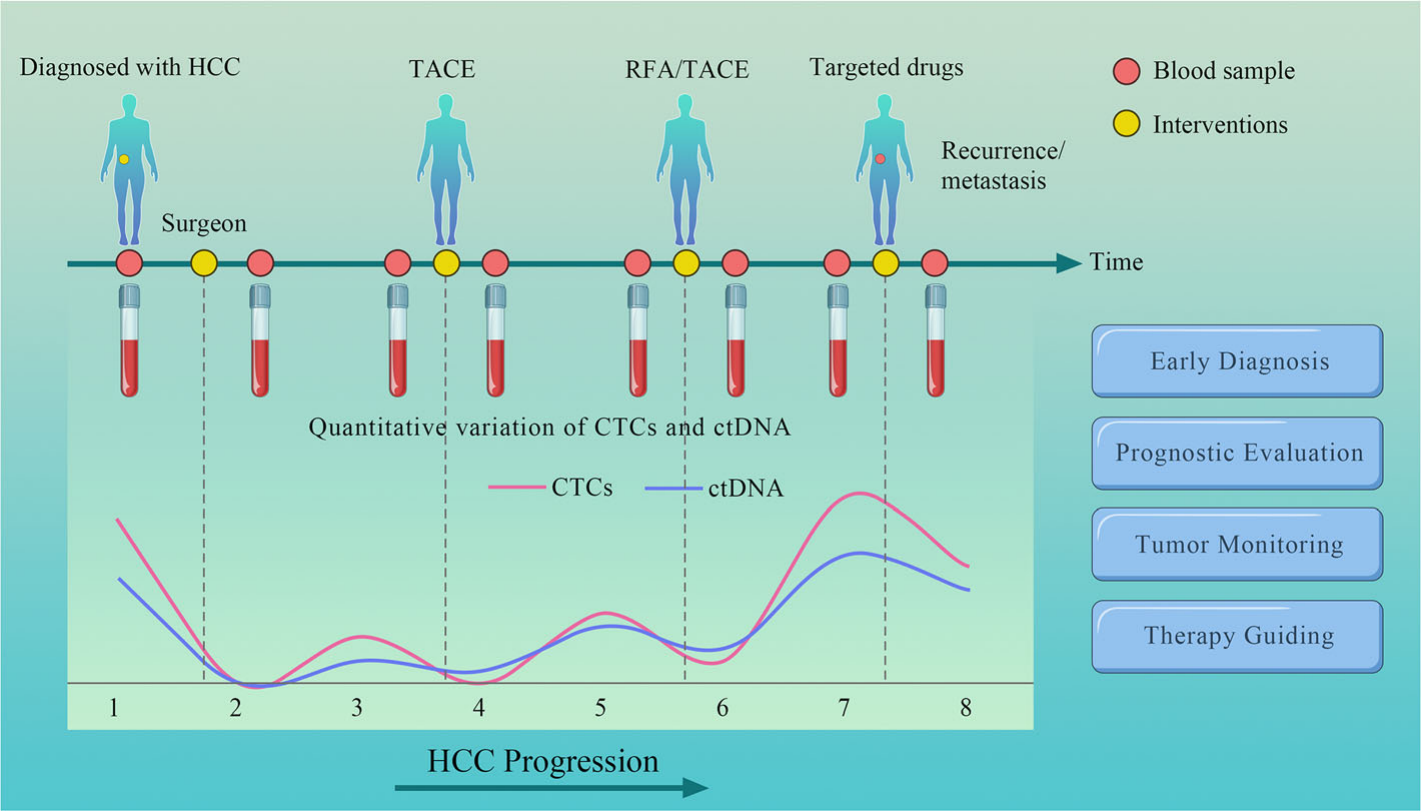
Phantom drawing of clinical applications of liquid biopsy in HCC patients. Monitoring the response and relapse of HCC patients using liquid biopsy, the levels of ctDNA and CTCs correlate well with HCC progression as well as various therapies, including surgical resection, TACE, radiofrequency ablation (RFA) and targeted molecular therapy

Moreover, somatic mutations detected in ctDNA can guide therapy. Ikeda et al. [54] evaluated 14 patients with advanced HCC. A patient with a CDKN2A-inactivating and a CTNNB1-activating mutation received palbociclib (CDK4/6 inhibitor) and celecoxib (COX-2/Wnt inhibitor) treatment and found low levels of AFP at baseline at 2 months. Another patient with a PTEN-inactivating and a MET-activating mutation received sirolimus (mechanistic target of rapamycin inhibitor) and cabozantinib (MET inhibitor) and found AFP declined by 63% (8320 to 3045 ng/mL). In conclusion, ctDNA derived from noninvasive blood tests can provide exploitable genomic profiles in patients with HCC and guide therapy in some ways (Fig. 2).

### Challenges and perspectives

In general, liquid biopsy is an exciting method that is noninvasive, overcomes tumor heterogeneity and can monitor tumor progression, recurrence or therapeutic response in real time. There is also an ongoing clinical trial (NCT02973204) from the US National Laboratory of Medicine (NIH) for CTCs and ctDNA in HCC, aiming at predicting which patients require special monitoring and individualized therapy and exploring the value of these tests in supporting clinical decision-making. However, there are challenges that researchers face in translating liquid biopsy from bench to bedside that still stand in the way.

First, the biological basis of CTCs or ctDNA is controversial; for example, the exact mechanisms by which ctDNA is released into the blood remains to be clarified. Apoptosis and necrosis are the most discussed origins of cfDNA [112]. Autophagy and hypoxia might account for ctDNA release. Malignant cells could activate autophagy as a survival mechanism, and autophagic activity regulates apoptosis; as a result, ctDNA is released [113]. Exposures to intermittent hypoxia (IH) could increase the shedding of ctDNA into the circulation [114]. Therefore, it is important to gain a better understanding of the biological characteristics of the liquid biopsy.

The descriptions of CTCs and ctDNA in this review are mainly focused on HCC. However, many other types of cancers may detect the same mutations in genes, such as TP53, KRAS, or BRAF. Cancer-associated mutations occur with increasing age, raising a problem that cannot be neglected: how could cfDNA be tissue-specific? The epigenetic biomarkers of cfDNA may answer this question [115]. DNA methylation is the most favored epigenetic modification whose profile is highly tissue-specific and shows the potential to determine the tissue origin of cfDNA [116–119]. In addition, the nucleosome occupancy and the ending pattern of cfDNA molecules also reflect the original tissues. By deep sequencing cfDNA, Snyder et al. generated maps of genome-wide in vivo nucleosome occupancy and found nucleosome spacing patterns, providing the information of the tissues of origin for cfDNA [120]. There were millions of plasma ctDNA end coordinates across the genome, and HCC-associated ctDNA had preferred end signatures different from other cfDNA [121], making it possible to trace the origin of ctDNA.

Moreover, liquid biopsy, especially ctDNA, shows considerable potential in classifying molecular subtyping of certain malignancies. Plasma ctDNA genotyping can classify transcriptionally defined tumor subtypes of diffuse large B cell lymphoma (DLBCL). Biopsy-free plasma genotyping of ctDNA in 41 DLBCL patients was highly consistent with tumor tissue biopsy classification (88%) [122], which may facilitate individualized therapy. Martínez-Ricarte F et al developed a sequencing platform to simultaneously and rapidly genotype seven genes (IDH1, IDH2, TP53, TERT, ATRX, H3F3A, and HIST1H3B) in CSF ctDNA, allowing the subclassification of diffuse gliomas, the most common primary tumor of the brain, having different subtypes with diverse prognoses [123].

Nevertheless, liquid biopsy has its own limitations. The techniques of collecting “liquid”, and the isolation, enrichment or detection of CTCs and ctDNA must be standardized. Most of the current studies used different technologies or assays to detect CTCs or ctDNA, resulting in diverse sensitivity and specificity. Although ctDNA has high specificity in diagnosis, a multi-marker analysis may offer a more comprehensive insight into cancer specificity [55, 56, 101]. In addition, more multicenter, larger and longer-term studies are urgently needed for the implementation of the liquid biopsies clinically, including clinical trials. Currently, most of the data gathered have been within proof-of-concept studies and lack of credibility. Altogether, the liquid biopsy is a critical part of precise medicine, and is believed will become a clinical reality in near future.

## Data Availability

Not applicable.

## Abbreviations

AFP: Alpha-fetoprotein
AFU: α-L-fucosidase
AI: Allelic imbalance
APC: Adenomatosis Polyposis Coli
BCLC: Barcelona Clinic Liver Cancer
BEAMing: Bead, Emulsion, Amplification, Magnetic
Capp-Seq: Cancer personalized profiling by deep sequencing
CDK6: Cyclin-dependent kinase 6
cfDNA: Cell-free DNA
COX2: Cytochrome c oxidase subunit 2
CSF: Cerebrospinal fluid
CTCs: Circulating tumor cells
ctDNA: Circulating tumor DNA
CTNNB1: Catenin beta 1
DFS: Disease-free survival
dPCR: Digital PCR
EGFR: Epidermal Growth Factor Receptor
EMT: Epithelial-mesenchymal transition
EpCAM: Epithelial cell adhesion molecule
FISH: Fluorescence in situ hybridization
GSTP1: Glutathione S-transferase pi 1
HBV: Hepatitis B Virus
HCC: Hepatocellular carcinoma
HCV: Hepatitis C Virus
HER2: Human epidermal growth factor receptor 2
hTERT: Human telomerase reverse transcriptase
KRAS: Kirsten rat sarcoma viral oncogene homolog
miRNA: MicroRNA
NGS: Next-generation sequencing
OS: Overall survival
CDKN2A(p16INK4a): Cyclin dependent kinase inhibitor 2A
PSA: Prostate-specific antigen
PTEN: Phosphatase and tensin homolog
RASSF1A: Ras association domain family 1A
RT-PCR: Reverse transcription polymerase chain reaction
Safe-SeqS: Safe-Sequencing System
SFRP1: Secreted frizzled related protein 1
TACE: Transarterial chemoembolization
TAm-Seq: Tagged-amplicon deep sequencing
TEPS: Tumor-educated blood platelets
TERT: Telomerase reverse transcriptase
TP53: Tumor protein p53
WGS: Whole-genome sequencing
